# Comparative photoelastic study of dental and skeletal anchorages in the canine retraction

**DOI:** 10.1590/2176-9451.19.1.100-105.oar

**Published:** 2014

**Authors:** Cristiane Aparecida de Assis Claro, Rosana Villela Chagas, Ana Christina Elias Claro Neves, Laís Regiane da Silva-Concílio

**Affiliations:** 1 Assistant Professor, Department of Dentistry, University of Taubaté (UNITAU).; 2 Visiting Professor, Department of Dentistry, University of Taubaté (UNITAU).; 3 Assistant Professor, Department of Dentistry, University of Taubaté (UNITAU).

**Keywords:** Orthodontics, Tooth movement, Orthodontic anchorage, Procedures

## Abstract

**Objective:**

To compare dental and skeletal anchorages in mandibular canine retraction by means of a stress distribution analysis.

**Methods:**

A photoelastic model was produced from second molar to canine, without the first premolar, and mandibular canine retraction was simulated by a rubber band tied to two types of anchorage: dental anchorage, in the first molar attached to adjacent teeth, and skeletal anchorage with a hook simulating the mini-implant. The forces were applied 10 times and observed in a circular polariscope. The stresses located in the mandibular canine were recorded in 7 regions. The Mann-Whitney test was employed to compare the stress in each region and between both anchorage systems. The stresses in the mandibular canine periradicular regions were compared by the Kruskal-Wallis test.

**Results:**

Stresses were similar in the cervical region and the middle third. In the apical third, the stresses associated with skeletal anchorage were higher than the stresses associated with dental anchorage. The results of the Kruskal-Wallis test showed that the highest stresses were identified in the cervical-distal, apical-distal, and apex regions with the use of dental anchorage, and in the apical-distal, apical-mesial, cervical-distal, and apex regions with the use of skeletal anchorage.

**Conclusions:**

The use of skeletal anchorage in canine retraction caused greater stress in the apical third than the use of dental anchorage, which indicates an intrusive component resulting from the direction of the force due to the position of the mini-implant and the bracket hook of the canine.

## Abstract (pt)

**Objetivo:**

comparar as ancoragens dentária e esquelética na retração do canino inferior, por meio do estudo da distribuição de tensões.

**Métodos:**

foi confeccionado um modelo fotoelástico de segundo molar a canino, sem o primeiro pré-molar, e simulada a retração do canino inferior com elástico preso a dois tipos de ancoragem: dentária, no primeiro molar conjugado aos dentes adjacentes; e ancoragem esquelética, em gancho simulando o mini-implante. As forças foram aplicadas 10 vezes e observadas no polariscópio circular. As tensões no canino inferior foram registradas em 7 regiões. O teste de Mann-Whitney foi aplicado para comparar as tensões em cada região, considerando os dois sistemas de ancoragem. As tensões nas regiões perirradiculares do canino foram comparadas pelo teste de Kruskal-Wallis.

**Resultados:**

as tensões foram similares tanto na região cervical quanto no terço médio. No terço apical, as tensões associadas à ancoragem esquelética foram maiores que as tensões associadas à ancoragem dentária. Os resultados do teste de Kruskal-Wallis mostraram que as maiores tensões foram identificadas nas regiões cervicodistal, apicodistal e na região do ápice com o uso da ancoragem dentária; e com o uso da ancoragem esquelética, as maiores tensões se localizaram nas regiões apicodistal, apicomesial, cervicodistal e no ápice.

**Conclusão:**

o uso de ancoragem esquelética na retração promoveu maior tensão no terço apical do que o uso da ancoragem dentária, indicando um componente intrusivo devido à direção da força decorrente da posição do mini-implante e do gancho do braquete do canino.

## INTRODUCTION

The concern over anchorage has always accompanied the evolution of Orthodontics. Many resources have been used with the purpose of avoiding undesired movement of the anchoring unit, namely: headgear appliances, lingual arches and transpalatal bars. Some approaches to anchoring consider the biological basis and avoid mobility of posterior teeth before space closure. In these cases, rigid appliances are combined with monitoring of the occlusion in order to achieve anchoring.^1^ Strategies such as including the second molar in the mechanics, using low forces for retraction and low friction mechanics have already been suggested to minimize loss of anchorage.^2^ Despite the availability of several papers studying anchorage, due to methodological issues, the scientific evidence is not considered sufficient to identify the most effective anchoring system.^3^

When maximum anchorage is needed to achieve the proposed objectives, mini-implants have been adopted to replace dental anchorage. The efficiency of mini-implants in controlling loss of anchorage has been confirmed in a study^4^ that identified average anchorage loss of 1.6 mm in the maxilla and 1.7 mm in the mandible on the side where canine retraction was anchored in the molar, and no loss on the side with mini-implants. Skeletal anchorage has also been named absolute anchorage;^5,6^ however, some researchers have questioned this nomenclature because mini-implant movement and loss of anchorage have been identified even with the use of skeletal anchorage.^7,8^

Skeletal anchorage can be used in a direct or indirect manner. The indirect one does not influence the vector systems of the forces employed, however, if the mini-implant moves, it might result in loss of anchorage of the involved teeth. This possibility does not exist in direct anchorage; however, the location of mini-implants will directly influence the result of retraction movement. The terms high-pull or high installation (distance greater than 10 mm from the mini-implant to the orthodontic arch), medium-pull (8 to 10 mm) and low-pull (<8 mm)^9^ are appropriate for the maxilla, but difficult to interpret when referred to the mandible. Therefore, it has been suggested that the force vectors be described as intrusive, intermediate and extrusive according to their effect on the anterior region.^10^

As for issues concerning biomechanics, especially magnitude and direction of the force employed to retract the canines, the proposed hypothesis is that the force vector resulting from direct skeletal anchorage would have a more vertical direction due to the mini-implant being inserted more apically than the molar hook used for dental anchorage. Additionally, it is also due to the fact that the canine hook is positioned closer to the occlusal surface than the mini-implant is, even though the mini-implant is inserted as close as possible to the cervical region. This situation would probably result in an intrusive effect associated with the retraction movement accompanying the use of skeletal anchorage.

Therefore, the present study compared dental and skeletal anchorage in mandibular canine retraction by means of stress distribution analysis performed in the periradicular region of the tooth with the use of a photoelastic model.

## MATERIAL AND METHODS

A photoelastic model was built from the mandibular second molar to the canine without the first premolar in order to simulate its extraction. Initially, bands and frictional brackets, Roth prescription (Ovation/Dentsply GAC International, Bohemia, NY, USA) were bonded to the artificial teeth (B2-306/Kilgore-Nissin; Kilgore International, Coldwater, MI, USA) and a 0.021 x 0.025-in stainless steel wire ("A" Company, San Diego, CA, USA) was installed.

This set was positioned in a rectangular (30 x 50 x 10 mm) silicone mold (Polipox, São Paulo, Brazil) filled with GIII flexible epoxy resin (Polipox). The set was then transferred to a vacuum chamber (-600 mm Hg) in order to have air bubbles eliminated. After 30 minutes, the photoelastic model was removed from the vacuum chamber. Tests were conducted 72 hours later. The 0.021 x 0.025-in stainless steel wire was replaced by a segment of 0.019 x 0.025-in ("A" Company, San Diego, CA, USA) stainless steel wire.

As for dentoalveolar anchorage, teeth from second molar to second premolar were splinted with metallic ligature (0.25 mm, Morelli, Sorocaba, SP, Brazil), and the hook of the first molar was used as a support for the application of force for canine retraction.

To simulate skeletal anchorage, a hook was bonded to a metallic post attached to a metallic base used to avoid deflection. The model was bonded in such a way that the simulated mini-implant (hook) was positioned between the first molar and the second premolar, 8 mm away from the arch. The forces were applied 10 times to the photoelastic model, under two anchorage conditions: in the first molar attached to the adjacent teeth, and simulating the mini-implant. A dynamometer (Correx 250; Haag-Streit, Berne, Switzerland) was used to certify that all rubber bands (Morelli, Sorocaba, SP, Brazil) activations had a retraction force of 100 cN.

**Figure 1:**
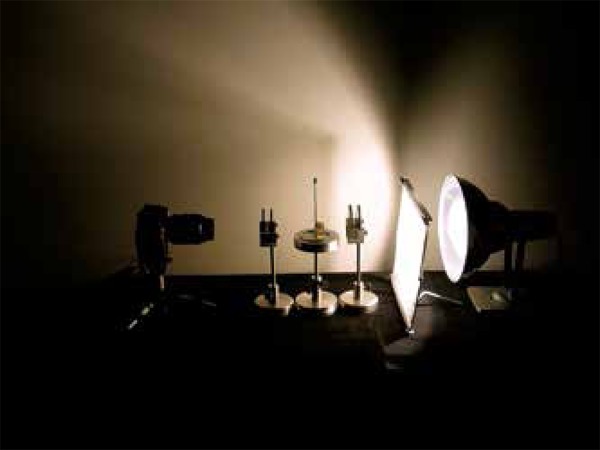
Circular polariscope.

The model was observed by means of a circular polariscope (Eikonal Instrumentos Ópticos, São Paulo, Brazil) assembled with the following components: light source (Photoflood 2), diffuser, polarizer, quarter wave plate, photoelastic model, quarter wave plate and polarizer (analyzer)^11^ (Fig 1). The circular polariscope was set up in a dark field, that is, the optical axes of the polarizer and the analyzer crossed to each other while the quarter wave plates crossed to each other at an angle of 45° with the polarizer and the analyzer.^12^ The photographic machine (D70 Nikon, Melville, NY, USA) was positioned in front of the analyzer and its settings remained throughout the experiment. The photoelastic model was positioned in a rotating platform previously marked to facilitate accurate placement of the model. The model was observed in the polariscope before forces were applied with the objective of verifying the absence of residual stress in the material. After force application, pictures were taken from the side view.

The fringe orders were verified around the canine, considering the sequence of colors produced in photoelastic material submitted to the increasing application of load and observation in the dark-field white-light polariscope (Table 1).^12^ It is possible to observe that values of fringe order and of relative delay increase with stress.

**Table 1:** Sequence of colors produced in a dark-field white-light polariscope. Source: ASTM D4093-95 (reapproved 2001) and www.vishay.com.

Color	Relative delay (δ) Nm	Fringe orderd δ/λ
Black	0	0
Gray	160	0.28
White	260	0.45
Light yellow	350	0.60
Orange	460	0.79
Intense red	520	0.90
Red-blue transition	577	1.00
Intense blue	620	1.06
Blue-green	700	1.20
Green- yellow	800	1.38
Orange	940	1.62
Pinkish red	1050	1.81
Red-green transition	1150	2.00
Green	1350	2.33
Green-yellow	1450	2.50
Red	1550	2.67
Red-green transition	1730	3.00
Green	1800	3.10
Pink	2100	3.60
Pink-green transition	2300	4.00
Green	2400	4.13

The absence of stress is shown in Figure 1A, while stress distribution associated with dental anchorage and skeletal anchorage is shown in Figures 1B and 1C, respectively.

## Statistical method

The significance level was set at 5% and adopted for all statistical tests. The error of the method was conducted to determine intra and interobserver agreement, for which the weighted kappa statistics was used. Ten photos from each group were reanalyzed by the same observer and by a second observer as well. To compare both types of anchorage, the Mann-Whitney test was used in each area evaluated, whereas to compare the stress between the periradicular regions of the canine, the Kruskal-Wallis test was used in each type of anchorage.

## RESULTS

The reliability of the values was confirmed by the error of method. The weighted kappa coefficients indicated that the agreements ranged from substantial to perfect. In the skeletal anchorage group, the coefficient ranged from 0.61 to 1.0 in the intraobserver, while it ranged from 0.61 to 0.88 in the interobserver analysis. In the dental anchorage group, the weighted kappa coefficients ranged from 0.61 to 1.0 in the intraobserver and from 0.61 to 0.78 in the interobserver analysis.

Table 2 shows the values of the median, first and third quartiles of the isochromatic fringe orders located in the canine radicular third in 7 regions: cervical-mesial (CM), cervical-distal (CD), middle-mesial (MM), middle-distal (MD), apical-mesial (AM), apical-distal (AD), and the apex (A). The Mann-Whitney test (with a significance threshold set at P < 0.05) was employed to compare the fringe orders in each region, considering both anchorage systems. The stresses on the cervical and middle third were similar (P > 0.05). In the apical third, the stresses associated with skeletal anchorage (medians: AM = 0.6, AD = 1.0, and A = 0.9) were significantly higher than the stresses associated with dental anchorage (medians: AM = 0.45, AD = 0.79, and A = 0.6) (P < 0.05).

**Table 2:** Median, first and third quartiles related to dental and skeletal anchorage, and results of Mann-Whitney comparisons between both groups in the areas evaluated.

Area	Dental anchorage			Skeletal anchorage			p-value
	Median	Q1	Q3	Median	Q1	Q3	
CM	0.28	0.28	0.28	0.28	0.28	0.28	>0.05
CD	0.90	0.79	0.90	0.79	0.79	0.90	>0.05
MM	0.28	0.28	0.28	0.28	0.28	0.28	>0.05
MD	0.00	0.00	0.00	0.00	0.00	0.00	>0.05
AM	0.45	0.45	0.45	0.60	0.60	0.60	0.0002
AD	0.79	0.79	0.90	1.00	0.90	1.06	0.0009
A	0.60	0.60	0.79	0.90	0.90	0.90	0.0003

CM = cervical-mesial CD = cervical-distal MM = middle-mesial MD = middle-distal AM = apical-mesial AD = apical-distal A = apex

Table 3 shows the comparison between the fringe orders originating from retraction force associated with the use of dental anchorage in the canine periradicular regions, which was performed via the Kruskal-Wallis test (significance at P < 0.05) . Higher stress concentrations were identified in the cervical-distal (0.9), apical-distal (0.79) and apex (0.6) regions. The stresses in these areas did not differ, but were significantly higher than in the cervical-mesial (0.28), middle-mesial (0.28) and middle-distal (0) regions. In the apical-mesial region (0.45), the stress was lower than in the cervical-distal and apical-distal, however, it was not statistically different from the apical region stress.

**Table 3:** Results of Kruskal-Wallis test for comparison between the areas with dental anchorage.

Area	Dental anchorage		
	Median	Middle rank	
CM	0.28	20	C/D
CD	0.9	61.6	A
MM	0.28	20	C/D
MD	0	6.5	D
AM	0.45	35.5	B/C
AD	0.79	57.6	A
A	0.6	47.3	A/B

Capital letters differ in the vertical direction.

Table 4 shows the comparison, via the Kruskal-Wallis test (significance set at P < 0.05), between the fringe orders in the canine periradicular regions originating from retraction force associated with the use of skeletal anchorage. The highest stresses were located in the apical-distal (1.0), apex (0.9), cervical-distal (0.79) and apical-mesial (0.6) regions. The stresses in these areas did not differ, but were significantly higher than in the cervical-mesial (0.28) and middle-distal (0) regions. In the middle-mesial region (0.28), the stress was lower than in the apical-distal (1.0), apex (0.9) and cervical-distal (0.79) regions, however, it did not statistically differ from the apical-mesial (0.6) region.

**Table 4:** Results of the Kruskal-Wallis test for comparison between areas with skeletal anchorage.

Area	Skeletal anchorage		
	Median	Middle rank	
CM	0.28	19	C
CD	0.79	47.6	A
MM	0.28	20.1	B/C
MD	0	7.4	C
AM	0.6	35.9	A/B
AD	1	63.7	A
A	0.9	54.8	A

Capital letters differ in the vertical direction.

## DISCUSSION

The method used to evaluate the effects of skeletal anchorage on orthodontic movements requires further research and development. In the present study, the hook attached to a metallic post assembled to a stand to which the model was bonded was an artifice that allowed the application of force and the simulation of skeletal anchorage (if the hook were simply bonded to the model, it could itself generate stress). The artifice adopted in this study is based on a research carried out by Nakamura et al^13^ who used a support external to the photoelastic model to simulate the application of distalization force to the mandibular molars anchored to mini-implants.

While the safest zone for the installation of mini-implants is located between the first and second molars in the mandible,^14^ the hook used to simulate the mini-implant positioned between the second premolar and the first molar in order to achieve direct skeletal anchorage.^10^

Generally, mini-implants are installed more apically than the molar hooks; therefore, retraction associated with direct anchorage of mini-implants tends to introduce a vector of force that is more intrusive than what is observed with the use of traditional mechanics.^10^ This statement is supported by the present study, given the fact that the use of skeletal anchorage promoted significantly higher stresses in the canine apical region than the use of dental anchorage. It is worth mentioning that since skeletal anchorage does not allow the dissipation of mechanical force during retraction, as it occurs in dental anchorage, it can justify the higher stress magnitude observed in the apical region where skeletal anchorage was used.

By using dental anchorage, the action line of the retraction force went farther from the center of resistance of the canine, which, in single-root teeth, is located at 33-42% of the distance between the alveolar crest and the root apex.^15^ Although there was no statistically significant difference between the types of anchorage in the cervical-distal region, the isochromatic fringes in this region, with the use of dental anchorage (Figure 1B), confirm that the canine retraction force tends to distally tip the crown when traction is anchored on the molars, even if 0.019 x 0.025-in wire and brackets with 7° of angulation are used. Inclination and extrusion of the canine occur in response to orthodontic wire deflection caused by distalization force and are also due to the inherent difficulty of the tooth in performing a genuine movement of radicular translation. Therefore, there was a distal tipping trend of the canine regardless of the anchorage system used. Conversely, as the force anchored in the mini-implant presented higher stress in the apical region, it is assumed that there was a greater control of that tipping and extrusion tendency (Fig 1C).

The intrusive component of force associated with the distalization force in skeletal anchorage, significantly increased the stress in the apical region in comparison to dentoalveolar anchorage (Table 2; AM, AD, and A regions).

With the use of dental anchorage, as shown in Table 3, the highest stresses were identified in the cervical-distal region (0.9). This stress value, however, does not significantly differ from the stress in the apical-distal region (0.79) or in the apical region (0.6). The observation that there was also stress in the apical region, even with the use of dental anchorage, can be explained by the type of bracket used in the canine. In Roth prescription brackets, the 7 ° angulation tends to transfer force to the apical region, especially in the distal face of the apex.

On the other hand, the use of skeletal anchorage, when comparing the stress between the canine periradicular regions, indicated that the highest stress magnitude was located in the apical-distal region (1.0), however, that value was not statistically significant different in relation to the apical (0.9), apical-mesial (0.6) and cervical-distal (0.79) regions (Table 4). Future photoelastic studies might simulate the different directions of traction, varying the positions of the mini-screws and also the height of the hook in the anterior region to compare the stresses generated by the different force systems.

Although satisfying results can be obtained with either skeletal or conventional anchorage, retraction with the use of mini-implants does not require patient collaboration,^16^ and it is undoubtedly an anchorage resource that is gaining followers in the orthodontic practice.

## CONCLUSION

Using skeletal anchorage for retraction promoted greater stress in the apical third in comparison to dental anchorage, which indicates an intrusive component originating from the force direction that results from the position of the mini-implant and the canine bracket hook.

## Figures and Tables

**Figure 2:**
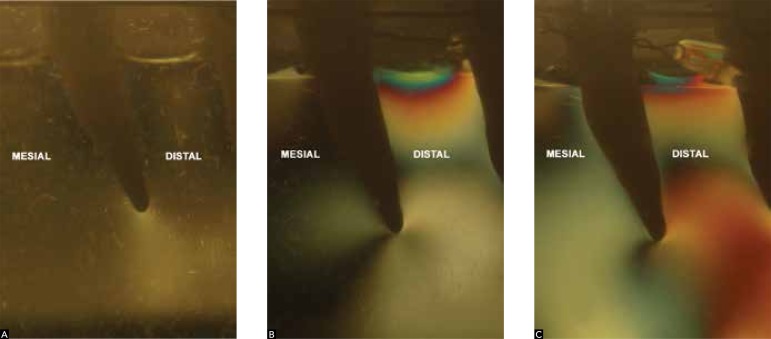
Visualization of stress in dark-field circular polariscope: A) absence of stress; B) stress distribution with dental anchorage and C) stress distribution with skeletal anchorage.
